# Phytochemical Profiling and Antioxidant and Enzymatic Evaluation of Extracts from the Antarctic Lichens *Polycauliona candelaria* and *Placopsis antarctica*

**DOI:** 10.3390/molecules31132242

**Published:** 2026-06-25

**Authors:** Alfredo Torres-Benítez, Nicolás Pizarro-Piña, Javier Romero-Parra, Gabriel Vargas-Arana, Marta Sánchez, María Pilar Gómez-Serranillos, Mario J. Simirgiotis

**Affiliations:** 1 Carrera de Química y Farmacia, Facultad de Ciencias, Universidad San Sebastián, General Lagos 1163, Valdivia 5090000, Chile; 2 Instituto de Farmacia, Facultad de Ciencias, Universidad Austral de Chile, Campus Isla Teja, Valdivia 5090000, Chile; nicolas.pizarro01@alumnos.uach.cl; 3 Departamento de Química Orgánica y Fisicoquímicas, Facultad de Ciencias Químicas y Farmacéuticas, Universidad de Chile, Olivos 1007, Santiago 8380544, Chile; javier.romero@ciq.uchile.cl; 4 Facultad de Industrias Alimentarias, Universidad Nacional de la Amazonía Peruana, Iquitos 16001, Peru; gabriel.vargas@unapiquitos.edu.pe; 5 Departamento de Farmacología, Farmacognosia y Botánica, Facultad de Farmacia, Universidad Complutense de Madrid, Plaza Ramón y Cajal s/n, Ciudad Universitaria, 28040 Madrid, Spain; martas15@ucm.es (M.S.); pserra@ucm.es (M.P.G.-S.)

**Keywords:** chemical characterization, bioactive compounds, extracts, Antarctic lichens, screening biological, antioxidant activity, enzymatic inhibition

## Abstract

The high prevalence and incidence of neurodegenerative diseases pose a public health challenge and drive the search for alternative treatments. This study determined the chemical composition of hydroalcoholic extracts from the Antarctic species *Polycauliona candelaria* and *Placopsis antarctica* and evaluated their antioxidant and cholinesterase-inhibitory potential through in vitro assays and molecular docking. Using UHPLC/ESI/QToF/MS, 16 compounds were tentatively identified in *P. candelaria* and 11 in *P. antarctica*. *P. antarctica* exhibited greater antioxidant capacity (2.69 ± 0.15 mg GAE/g in TPC, and an IC_50_ for DPPH and ABTS of 330.64 ± 0.02 and 63.33 ± 0.02 µg/mL, respectively) and inhibitory activity (IC_50_ for AChE and BuChE of 654.42 ± 0.03 and 845.58 ± 0.01 µg/mL, respectively) similar to *P. candelaria*. Molecular docking analyses revealed that gyrophoric acid and stictic acid possess outstanding binding affinities, comparable to the drug galantamine, by effectively interacting with the catalytic sites of the enzymes. This is the first report on the chemical compounds present in extracts of *P. antarctica* and *P. candelaria* and contributes to the understanding of their therapeutic potential.

## 1. Introduction

Neurodegenerative diseases represent a critical challenge for global public health, driven by population aging [1,2]. At the pathological level, oxidative stress is recognized as a key factor in the progression of neurodegenerative diseases, acting as an active driver of neuronal damage [3,4]. This process arises from an imbalance between the production of reactive oxygen and nitrogen species and the organism’s antioxidant capacity, leading to lipid peroxidation, protein oxidation, and DNA damage [4,5]. In the central nervous system, this susceptibility is exacerbated by high oxygen consumption and the abundance of readily oxidizable lipids, thereby promoting the accumulation of reactive compounds that disrupt cellular function [4]. In Alzheimer’s disease, this pro-oxidant environment promotes the accumulation of β-amyloid peptides and mitochondrial dysfunction [3,5], whereas in Parkinson’s disease, it contributes to the degeneration of dopaminergic neurons through mechanisms associated with oxidative stress and alterations in dopamine metabolism [3,4].

Given the multifactorial complexity of neurodegenerative diseases and the limitations of current pharmacological therapies, research has increasingly prioritized the exploration of natural products as a source of bioactive compounds with therapeutic potential, capable of providing valuable insights for the development of new drugs aimed at delaying disease onset or alleviating symptoms [6,7,8]. In this context, chronic oxidative stress is recognized as one of the main factors associated with neuronal deterioration, which has driven growing interest in natural antioxidants capable of counteracting this imbalance [9,10,11,12]. Among these compounds, polyphenols, flavonoids, and other phytochemicals stand out, as they not only act as direct scavengers of reactive species but also modulate cellular mechanisms involved in redox balance, inflammation, and mitochondrial function [12,13,14].

Within this framework, lichens have emerged as a promising source of bioactive compounds with potential neuroprotective and antioxidant properties. These organisms result from a symbiotic association between a fungus (mycobiont) and one or more photosynthetic partners (photobionts), such as green algae or cyanobacteria. This relationship enables the production of unique secondary metabolites that are rarely found in other biological systems [15,16]. Their remarkable ability to colonize and persist in extreme environments—including Antarctic regions, alpine zones, tropical habitats, and deserts—has driven the evolution of specialized biosynthetic pathways that yield structurally diverse compounds, predominantly of phenolic nature, whose production is enhanced by harsh environmental conditions [17]. These compounds exhibit a wide range of biological activities, including cytotoxic, anti-inflammatory, and notably strong antioxidant effects [18,19,20]. In terms of neuroprotective activity, species such as *Cetraria islandica* and *Vulpicida canadensis* are key subjects of study due to their high phenolic content and ability to reverse oxidative damage [21,22]. Likewise, compounds such as evernic, fumarprotocetaric, lecanoric, stictic, and usnic acids, found in species of the genera *Ramalina*, *Evernia*, and *Hypogymnia*, exhibit optimal antioxidant capabilities and provide neuronal protection against the loss of dopaminergic neurons [23,24].

**Figure 1:**
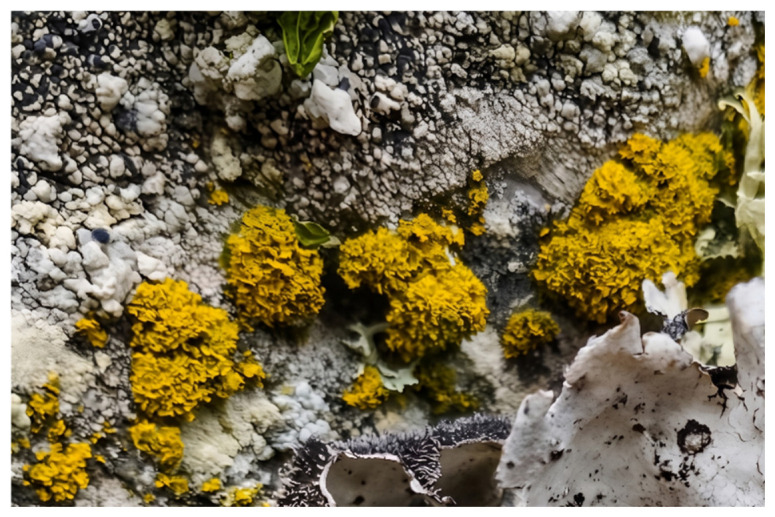
Lichen thallus of *Polycauliona candelaria*.

**Figure 2:**
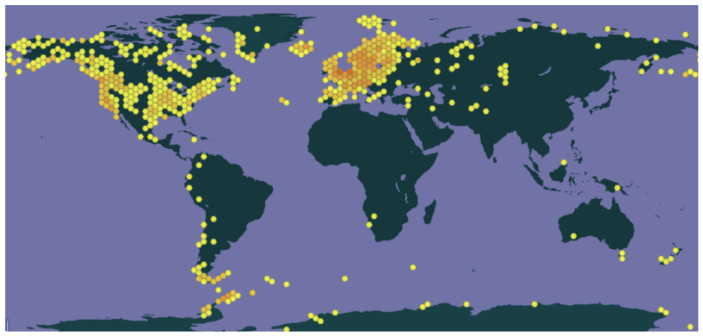
Distribution of the lichen *Polycauliona candelaria*. GBIF.

One of the lichens of interest for this research is *Polycauliona candelaria* (formerly known as *Xanthoria candelaria*), a foliose to subfruticose lichen belonging to the family Teloschistaceae, distinguished by its characteristic yellow–orange coloration. It typically forms small cushion-like structures measuring 2–3 cm in width, which often coalesce into more extensive colonies. The thallus is composed of thin, partially cylindrical lobes with a wrinkled surface, while the lower surface exhibits a pale whitish-yellow coloration (Figure 1). A distinctive feature of this species is the presence of granular blastidia, which confer a spiculated appearance. *P. candelaria* is a nitrophilous species that commonly grows on nutrient-enriched substrates, particularly rocks influenced by bird droppings, and it exhibits a cosmopolitan distribution ranging from temperate regions to polar environments in both hemispheres [25,26] (Figure 2).

**Figure 3:**
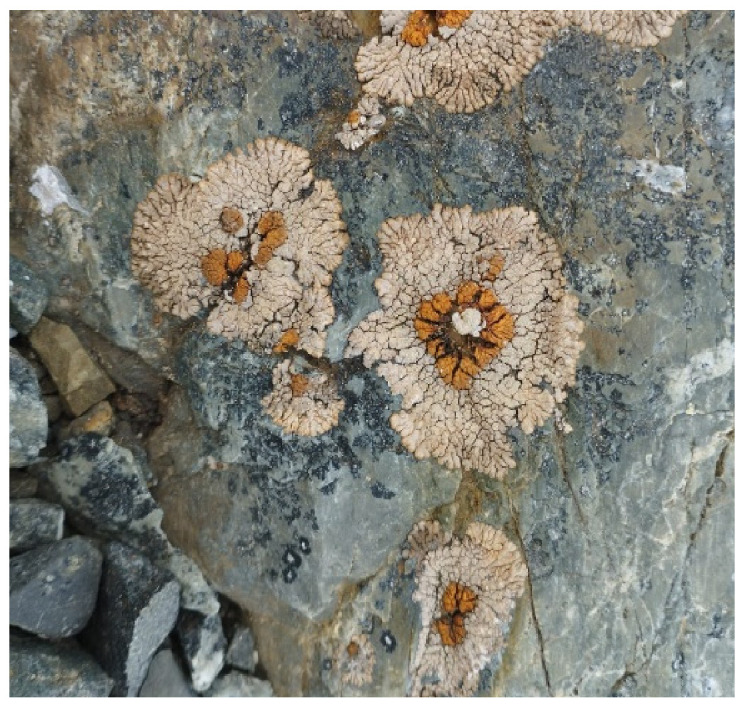
Lichen thallus of *Placopsis antarctica*.

**Figure 4:**
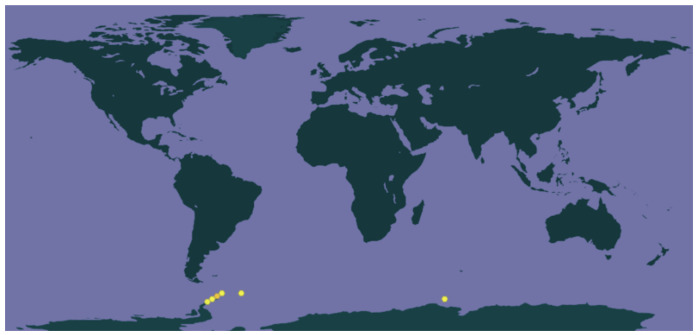
Distribution of the lichen *Placopsis antarctica.* GBIF.

On the other hand, *Placopsis antarctica* is a crustose lichen belonging to the family Trapeliaceae, characterized by forming circular patches firmly attached to rock surfaces through a compact external cortex and an internal medulla, with well-defined marginal lobes. Its most distinctive feature is the presence of cephalodia, small wart-like pink to violet-brown structures containing nitrogen-fixing cyanobacteria, which enable this species to thrive even in nutrient-poor substrates (Figure 3). *P. antarctica* is a nitrophilous species endemic to Maritime Antarctica and subantarctic regions (Figure 4), where it commonly grows on ice-free coastal rocks, frequently in sites enriched by seabird guano [27,28].

Despite the chemical richness of polar lichens, many species remain unstudied, and this study focuses on *P. candelaria* and *P. antarctica*, two nitrophilic species found in Maritime Antarctica. The novelty of this work lies in being the first detailed report on the chemical composition and biological activities (antioxidant and cholinesterase-inhibitory) of these two species, thereby contributing to the assessment of Antarctic lichens as sources of molecules to combat oxidative stress and cholinergic deficiency. The objective of this study was to determine the chemical composition of hydroalcoholic extracts from the Antarctic lichens *Polycauliona candelaria* and *Placopsis antarctica* and to assess their antioxidant and enzyme-inhibitory potential through in vitro and molecular docking assays.

## 2. Results and Discussion

### 2.1. Metabolomics Profiles of Lichen Extracts

#### 2.1.1. Chromatographic Analysis of *Polycauliona candelaria*

**Figure 5:**
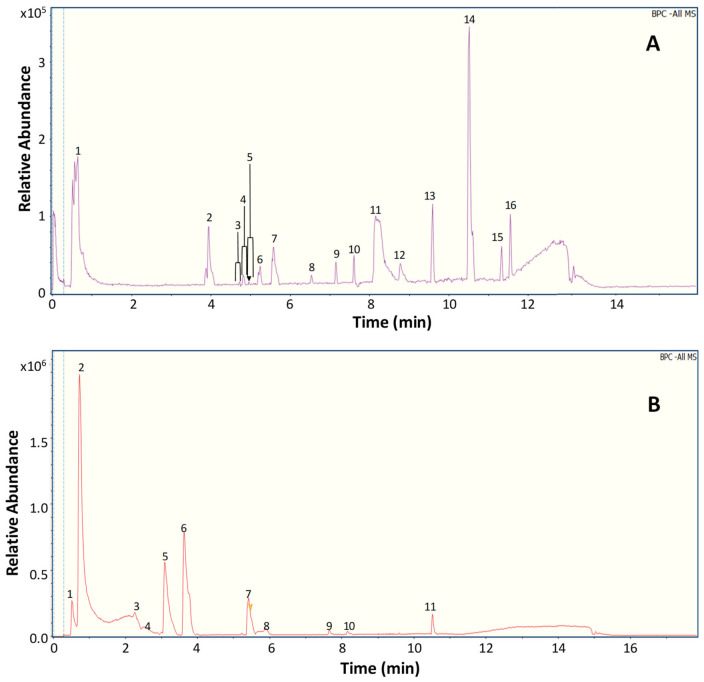
Chromatogram of the hydroalcoholics extracts obtained by UHPLC-ESI-QToF-MS. (**A**) *P. candelaria*; (**B**) *P. antarctica.* Note: the numbering of the peaks in each chromatogram corresponds to the different compounds listed in Table 1 and Table 2.

Analysis of the compounds present in the hydroalcoholic extract of *P. candelaria* using UHPLC/ESI/QToF/MS with negative ionization allowed for the tentatively identification of sixteen compounds, which were classified as organic acids, sugar alcohols, phenolic derivatives, anthraquinones, carotenoids, fatty acids, and dibenzofurans (Figure 5A and Table 1).

Organic acid: one organic acid was tentatively identified in the peak 1 as 2-isopropylmalic acid (C_7_H_11_O_5_) with a molecular anion at *m*/*z* 181.0686 and diagnostic peak at *m*/*z* 291.0039.

Sugar alcohol: one sugar alcohol was tentatively identified in the peak 2 as mannitol (C_6_H_13_O_6_) with a molecular anion at *m*/*z* 191.05592 and diagnostic peak at *m*/*z* 127.03968.

Phenolic derivative: one phenolic derivative was tentatively identified in the peak 5 as 3-acetyl benzoic acid (C_9_H_7_O_3_) with a molecular anion at *m*/*z* 163.0375 and diagnostic peak at *m*/*z* 112.9839.

Anthraquinone: one anthraquinone was tentatively identified in the peak 8 as emodin (C_15_H_9_O_5_) with a molecular anion at *m*/*z* 269.0409 and diagnostic peaks at *m*/*z* 112.9841 and 225.05614.

Carotenoid: one carotenoid was tentatively identified in the peak 9 as 13-hydroxy-8′-apo-beta-caroten-8′-al (C_30_H_39_O_2_) with a molecular anion at *m*/*z* 431.3103 and diagnostic peak at *m*/*z* 112.9839.

Fatty acids: Five fatty acids were tentatively identified. The peak 10 as hexadecatetraenoic acid (C_16_H_23_O_2_), peak 13 as alpha linoleic acid (C_18_H_29_O_2_), peak 14 as linoleic acid (C_18_H_31_O_2_), peak 15 as palmitic acid (C_16_H_31_O_2_) and peak 16 as oleic acid (C_18_H_33_O_2_), with diagnostic peaks at *m*/*z* 175.1480, 259.0572, 112.9837, 116.9267 and 116.9264, respectively.

Dibenzofurans: The peak 11 was identified as usnic acid (C_18_H_15_O_7_) with a molecular anion at *m*/*z* 343.0779. The peak 12 was identified as usnic acid isomer (C_18_H_15_O_7_) with a molecular anion at *m*/*z* 343.0781.

Additionally, four compounds were identified as nitrogen-containing derivatives, which share fragmentation patterns with the identified phenolic and anthraquinone derivatives.

#### 2.1.2. Chromatographic Analysis of *Placopsis antarctica*

Analysis of the compounds present in the hydroalcoholic extract of *P. antarctica* using UHPLC/ESI/QToF/MS with negative ionization allowed for the tentatively identification of eleven compounds, which were classified as organic acids, phenolic acids, depsides, erythritol ester, depsidone, tridepside and glycolipid (Figure 5B and Table 2).

Organic acid: one organic acid was tentatively identified in the peak 1 as gluconic acid (C_6_H_11_O_7_) with a molecular anion at *m*/*z* 195.0488 and diagnostic peak at *m*/*z* 112.9843.

Phenolic acids: Two phenolic acids were tentatively identified. The peak 2 as orsellinic acid (C_8_H_7_O_4_) with a molecular anion at *m*/*z* 167.0330 and diagnostic peak at *m*/*z* 123.0437, and the peak 5 as orsellinic acid derivative with a molecular anion at *m*/*z* 167.03485 and diagnostic peak at *m*/*z* 149.02432.

Depsides: The peak 3 was tentatively identified as lecanoric acid derivative (C_16_H_13_O_7_) with a molecular anion at *m*/*z* 331.0421 and diagnostic peak at *m*/*z* 317.06558. The peak 8 was identified as lecanoric acid (C_16_H_13_O_7_) with a molecular anion at *m*/*z* 317.0628 and diagnostic peaks at *m*/*z* 285.0734 and 149.0243.

Erythritol ester: The peak 4 was tentatively identified as montagnetol derivative (C_16_H_21_O_9_) with a molecular anion at *m*/*z* 357.0578 and diagnostic peak at *m*/*z* 167.0327.

Depsidone: one depsidone was tentatively identified in the peak 6 as stictic acid (C_19_H_13_O_9_) with a molecular anion at *m*/*z* 385.0483 and diagnostic peak at *m*/*z* 297.0724.

Tridepside: one tridepside was tentatively identified in the peak 7 as gyrophoric acid (C_24_H_19_O_10_) with a molecular anion at *m*/*z* 467.0976 and diagnostic peak at *m*/*z* 317.06558.

Glycolipid: one glycolipid was tentatively identified in the peak 10 as heptadecyl D-glucoside (C_23_H_45_O_6_) with a molecular anion at *m*/*z* 489.3426 and diagnostic peak at *m*/*z* 279.2280.

Additionally, two compounds were identified as unknown; however, based on their mass fragmentation and diagnostic ion patterns, peak 9 could correspond to a triterpenoid derivative, and peak 11 to a phenolic derivative linked to a hydrocarbon or lipid chain.

**Table 2:** Identification by UHPLC/ESI/QToF/MS of the compounds present in the hydroalcoholic 154 extract of *P. antarctica*.

Peak	Retention Time (min)	Tentative Identification	Molecular Formula	Theoretical Mass ([M − H]^−^ *m*/*z*)	Measured Mass ([M − H]^−^ *m*/*z*)	Accuracy (ppm)	Metabolite Type	MS/MS Ions
1	0.3	Gluconic acid	C_6_H_11_O_7_	195.0510	195.0488	9.6	Organic acid	112.9843
2	0.7	Orsellinic acid	C_8_H_7_O_4_	167.03389	167.0330	5.78	Phenolic acid	123.0437
3	2.3	Lecanoric acid derivative	C_16_H_13_O_7_	331.04484	331.0421	3.5	Depside	317.06558
4	2.4	Montagnetol derivative	C_16_H_21_O_9_	357.0649	357.0578	1.85	Erythritol ester	167.0327
5	3.1	Orsellinic acid derivative	C_8_H_7_O_4_	167.03389	167.03485	5.78	Phenolic acid	149.02432
6	3.7	Stictic acid	C_19_H_13_O_9_	385.0412	385.0483	9.6	Depsidone	297.0724
7	5.4	Gyrophoric acid	C_24_H_19_O_10_	467.0973	467.0976	0.7	Tridepside	317.06558
8	5.9	Lecanoric acid	C_16_H_13_O_7_	317.0640	317.0628	1.45	Depside	285.0734, 149.0243
9	7.8	Triterpenoid derivative	C_30_H_41_O_11_	577.2688	577.2686	7.4	-	232.92471
10	8.2	Heptadecyl D-glucoside	C_23_H_45_O_6_	489.3421	489.3426	0.99	Glycolipid	279.2280
11	10.5	Phenolic derivative	C_16_H_23_O_4_	279.3543	279.2292	1.95	-	167.0327, 112.9838

Among the metabolites found in the species *P. candelaria* and *P. antarctica*, we identified depsides, dibenzofurans, depsidones, and aromatic derivatives, which are part of the reported diversity of lichen polyketides [29]. Similarly, the presence of fatty acids, organic acids, anthraquinones, and carbohydrates in *P. candelaria* and *P. antarctica* has also been identified in Antarctic lichens such as *Sphaerophorus globosus*, *Lecania brialmontii*, *Pseudephebe pubescens*, *Cladonia chlorophaea*, *Cladonia gracilis*, *Psoroma antarcticum*, *Psoroma hypnorum, Placopsis contortuplicata*, *Ochrolechia frigida*, *Umbilicaria antarctica* [30,31], *Himantormia lugubris* [32], and *Gondwania regalis* [22]. In the chemical profile of *P. antarctica* and *P. contortuplicata* [31], which belong to the same genus, they share only the compound lecanoric acid, and differences are evident that may be attributed to the type of extract evaluated for each species and inherent processes in the biosynthetic pathways.

Regarding the identification results, it should be noted that these did not depend solely on the exact mass, but rather on the integration of high-resolution mass, the isotopic pattern (mSigma), and a similarity of more than 85% with the fragmentation patterns recorded in the RIKEN library. Most compounds exhibited diagnostic fragments found in studies of Antarctic lichens, and although some precision errors exceed 5 ppm, all remain within the 20 ppm limit established by Metaboscape 4.0 software for processing complex biological matrices, ensuring internal consistency through the use of sodium formate as a calibrator.

On the other hand, based on previous in silico analyses of the interaction between lichen compounds and cholinesterase enzymes, six compounds of biological interest were selected for this study; their tentative identification is supported by their respective fragmentation patterns (Figures S1–S6): emodin, and linoleic, gluconic, orsellinic, stictic and gyrophoric acids.

### 2.2. Total Phenolic Contents and Antioxidant Activity

According to Table 3, *P. candelaria* had a low total phenolic content (0.56 ± 0.02 mg GAE/g) compared to *P. antarctica*, which exhibited a higher oxygen radical scavenging capacity (513.7 ± 8.8 µmol Trolox/g). Particularly in the ABTS assay, the lichen *P. antarctica* exhibited high antioxidant capacity with an IC_50_ of 63.33 ± 0.02 µg/mL, comparable to the standards gallic acid and quercetin. Variability was observed among the assays because they measure different chemical properties of the extracts. The FRAP assay measures exclusively the reducing capacity of the compounds through single-electron transfer (SET). The ORAC assay measures the ability of metabolites to neutralize peroxyl radicals via the hydrogen atom transfer (HAT) mechanism, while the DPPH and ABTS assays operate via mixed mechanisms (SET and HAT), although the ABTS assay is more versatile as it applies to both aqueous and lipophilic media. The behavior of the extracts in these assays revealed differences that may be primarily due to variations in total phenolic content, particularly the higher presence of depsides and depsidones in *P. antarctica*, which are more efficient hydrogen donors due to their highly substituted aromatic structures.

The low phenolic content found in *P. candelaria* is similar to that reported for *L. brialmontii* and *P. pubescens* [30], and for *P. antarctica*, it is comparable to that of *S. globosus* [30] and aqueous extracts of *Cladonia furcata* and *Hypogymnia physodes* [33]. Variability in antioxidant activity has been observed in extracts from multiple species and isolated bioactive compounds [34]. It is noteworthy that the low DPPH radical scavenging activity of the hydroalcoholic extract of *P. candelaria* is also evident in the aqueous, hydroalcoholic, methanolic, acetone, dichloromethane, and hexane extracts (4830 ± 1.97 to >28,000 µg/mL) of *Evernia prunastri* [35,36], the acetone extract of *Ramalina farinacea* (>1000 µg/mL) [37], and the ethanol extract of *Gondwania regalis* (2246.149 ± 0.086 µg/mL) [22].

### 2.3. Enzyme Inhibition Activity

Table 4 shows the inhibitory effects of hydroalcoholic extracts on cholinesterase enzymes. *P. candelaria* showed greater efficacy against the AChE enzyme, with IC_50_ values of 471.67 ± 0.04 µg/mL, while *P. antarctica* performed better against BuChE, with an IC_50_ of 845.58 ± 0.01 µg/mL; however, this confirms the variable results of lichen extracts in biological activity assays.

The inhibitory efficiency of the hydroalcoholic extracts of *P. candelaria* and *P. antarctica* was lower than the values reported for Antarctic species such as *L. brialmontti*, *P. pubescens*, *S. globosus* [30] and *Himantormia lugubris* [32], at concentrations below 60 µg/mL for AChE and BuChE, as well as compared to the ethyl acetate extracts of *Evernia prunastri* (AChE = 1.26 ± 0.03/BuChE = 1.70 ± 0.09 µg/mL) [38], the acetone extract (AChE = 3.1 ± 1. 2/BuChE = 9.8 ± 1.9 µg/mL) and methanolic (AChE = 3.9 ± 1.0/BuChE = 7.2 ± 0.7 µg/mL) extracts of *Cetraria islandica* [39] and the hexane and methanolic extracts of *Cladonia portentosa* [40].

### 2.4. Docking Molecular Studies

**Figure 6:**
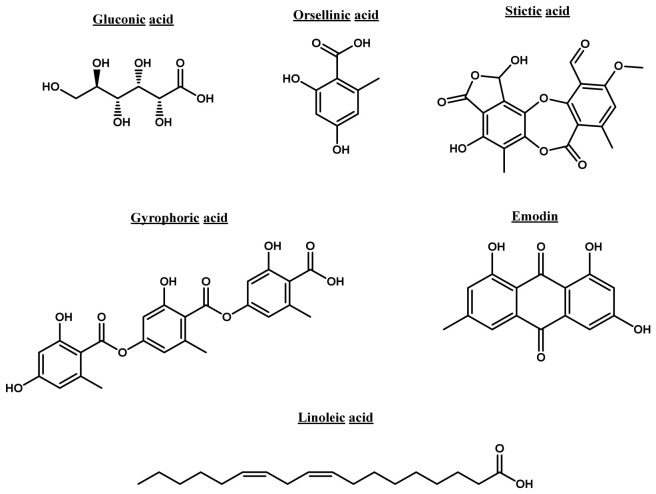
Gluconic acid, orsellinic acid, stictic acid, gyrophoric acid, emodin and linoleic acid evaluated by molecular docking at the catalytic site of acetylcholinesterase (*Tc*AChE) and butyrylcholinesterase (*h*BuChE).

Acetylcholinesterase (*Tc*AChE) and butyrylcholinesterase (*h*BuChE) are involved in cholinergic neurotransmission, with acetylcholinesterase playing the primary role. Owing to their catalytic efficiency and well-characterized three-dimensional structures, these enzymes enable the selective recognition and stabilization of small molecules through a combination of hydrogen bonding, π–π stacking, and hydrophobic interactions. In this context, docking studies provide valuable insights into how chemical scaffolds interact with key regions of the enzymes. Therefore, docking simulations were performed for gluconic acid, orsellinic acid, stictic acid, gyrophoric acid, emodin, and linoleic acid to rationalize their inhibitory activity and explore their binding behavior at the molecular level in both catalytic sites (Figure 6). Table 5 shows the binding energies for all mentioned compounds.

#### 2.4.1. Acetylcholinesterase (TcAChE) Docking Results

**Figure 7:**
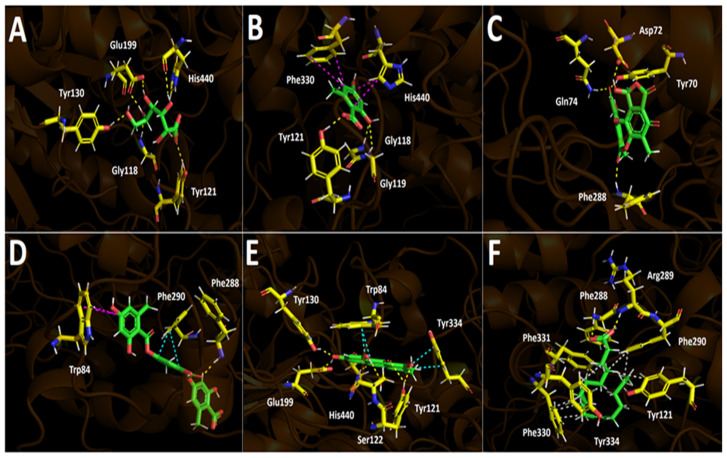
Predicted binding modes and intermolecular interactions of selected compounds with residues in the catalytic site of *Torpedo californica* acetylcholinesterase (*Tc*AChE). Yellow dotted lines indicate hydrogen bond interactions; cyan dotted lines indicate π–π interactions; magenta dotted lines indicate T-shaped interactions; and grey dotted lines indicate hydrophobic interactions. (**A**) Gluconic acid in the catalytic site; (**B**) orsellinic acid in the catalytic site; (**C**) stictic acid in the catalytic site; (**D**) gyrophoric acid in the catalytic site; (**E**) emodin in the catalytic site; (**F**) linoleic acid in the catalytic site.

Docking simulations at the *Tc*AChE catalytic site revealed a wide range of binding affinities among the metabolites from *P. antarctica* and *P. candelaria*. As shown in Table 5, gyrophoric acid and emodin exhibited the most favorable docking score values (−11.734 and −11.521 kcal/mol, respectively). Nonetheless, stictic acid can also be considered as a good *Tc*AChE inhibitor candidate, due to it exhibited a binding energy of −11.105 kcal/mol. The aforementioned values are highly comparable to the reference inhibitor galantamine which showed a binding energy of −11.749 kcal/mol. Analysis of the binding modes (Figure 7) indicates that the affinities of each compound are driven by distinct structural features.

Gluconic acid achieved a score of −8.833 kcal/mol through multiple hydrogen bonds with Gly118, Tyr 121, Tyr130, Glu199, and His440 (Figure 7A). Even the ability of this derivative to perform hydrogen bond interactions, its lack of aromatic moieties prevents the critical π-π stacking interactions required for a potent acetylcholinesterase inhibition. Thus, the absence of key hydrophobic interactions and the potential overestimation of polar stabilization are detrimental to the inhibitory activity of gluconic acid. Orsellinic acid showed a binding energy of −7.705 kcal/mol (Table 5). Its main interactions consist of four hydrogen bond interactions involving Gly118, Gly119, Tyr121, and His440. Furthermore, the benzene ring forms two T-shaped interactions with Phe330 and His440 (Figure 7B). Nevertheless, its relatively weak binding energy reflects a lower overall affinity, placing orsellinic acid among the one of the least favorable compounds.

As mentioned, stictic acid exhibited a favorable binding energy, owing to hydrogen bond interactions with Tyr70, Asp72, Gln74, and Phe288, as well as hydrophobic interactions with Trp279, Phe288, Phe290, Phe331, and Tyr334 (Figure 7C). Gyrophoric acid (Figure 7D) establishes a T-shaped interaction with Trp84, which could be taken as crucial for inhibition. Also it performs a π-π interaction with Phe290 and a hydrogen bonding with Phe288. Despite the relatively low number of specific polar contacts, the high binding affinity of gyrophoric acid may be attributed to its superior structural complementarity within the *Tc*AChE aromatic gorge, while the large hydrophobic surface of the depside scaffold maximizes van der Waals interactions.

Similarly, emodin (Figure 7E) demonstrates a robust stabilization within the aromatic gorge, forming π-π stacking interactions with Trp84 and Tyr334, complemented by hydrogen bonds with Tyr121, Ser122, Tyr130, Glu199 and His440. Finally, linoleic acid presented the lowest affinity in this series (−7.348$ kcal/mol), with its binding characterized by non-specific hydrophobic interactions across the aromatic gorge with Tyr121, Phe290, Phe330, Phe331, and Tyr334, as well as hydrogen bonds with Phe288 and Arg289 (Figure 7F).

#### 2.4.2. Butyrilcholinesterase (hBuChE) Docking Results

For *h*BuChE, binding energies were generally less favorable than those observed for *Tc*AChE, with the notable exception of stictic acid (−10.529 kcal/mol) and emodin (−9.470 kcal/mol), both of which outperformed the reference galantamine (−7.458 kcal/mol) for this specific enzyme. In contrast, the highly polar gluconic acid and the aliphatic chain of linoleic acid showed the weakest affinities of −4.527 and −7.829 kcal/mol, respectively.

**Figure 8:**
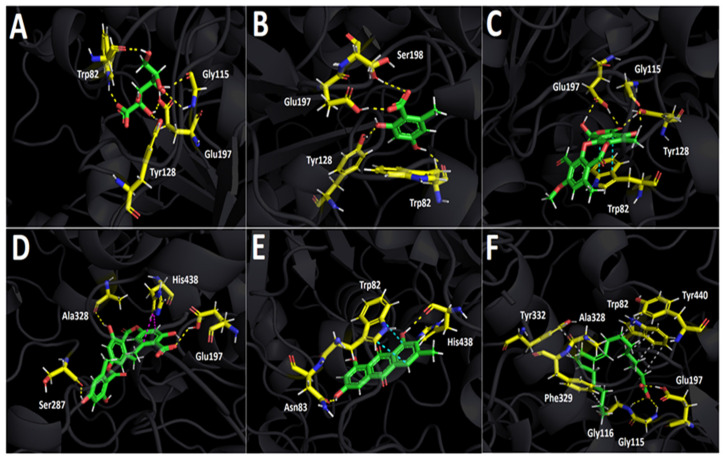
Predicted binding modes and intermolecular interactions of selected compounds with residues in the catalytic site of human butyrylcholinesterase (*h*BuChE). Yellow dotted lines indicate hydrogen bond interactions; cyan dotted lines indicate π–π interactions; magenta dotted lines indicate T-shaped interactions; and grey dotted lines indicate hydrophobic interactions. (**A**) Gluconic acid in the catalytic site; (**B**) orsellinic acid in the catalytic site; (**C**) stictic acid in the catalytic site; (**D**) gyrophoric acid in the catalytic site; (**E**) emodin in the catalytic site; (**F**) linoleic acid in the catalytic site.

Gluconic acid shows six hydrogen bond interactions with the residues Trp82, Gly115, Tyr128, and Glu197 (Figure 8A). Nonetheless, its lack of aromatic moieties prevents it from forming the interactions required for potent butyrylcholinesterase inhibition. Orsellinic acid, which is also a polar derivative, forms four hydrogen bonds with the amino acid residues Trp82, Tyr128, Glu197, and Ser198. However, no π–π interactions are observed for this compound, which may be detrimental to achieving a favorable binding energy (Figure 8B).

As mentioned, stictic acid, which is the compound with the most favorable binding energy profile, exhibits distinctive features in its interaction descriptors. In particular, it is characterized by its ability to occupy the larger catalytic pocket of hBuChE, forming π–π stacking interactions with Trp82 and four hydrogen bonds with Gly115, Tyr128, and Glu197 (Figure 8C). On the other hand, gyrophoric acid, despite being a bulky depside, fits within the catalytic gorge, forming a T-shaped interaction with His438 and hydrogen bonds with Glu197, Ser287, and Ala382 (Figure 8D), resulting in a binding energy of −8.777 kcal/mol (Table 5).

Emodin showed strong complementarity, establishing π–π interactions with Trp82 and hydrogen bonds with His438 and Asn83 (Figure 8E). Therefore, it is plausible that interactions with Trp82 (the hBuChE equivalent of TcAChE Trp84), are essential for stabilizing ligands within the choline-binding pocket. Finally, the less favorable binding energy of linoleic acid in hBuChE compared to *Tc*AChE suggests that its flexible hydrocarbon chain lacks sufficient specific anchoring points within the butyrylcholinesterase gorge. Indeed, this compound exhibits hydrophobic interactions with Trp82, Ala328, Phe329, Tyr332, and Tyr330, as well as hydrogen bonds with Gly115, Gly116, and Glu197 (Figure 8F).

**Figure 9:**
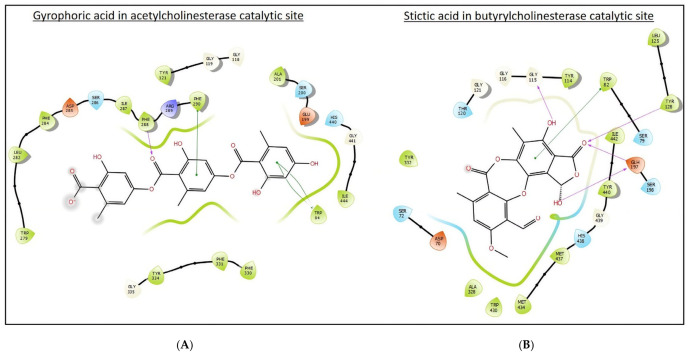
Two-dimensional diagrams illustrating the principal intermolecular interactions of gyrophoric acid (**A**) and stictic acid (**B**) within the catalytic site of acetylcholinesterase and butyrylcholinesterase, respectively. Key hydrogen bonding, π–π/T-shaped, and hydrophobic interactions with active-site residues are depicted. Purple arrows indicate hydrogen bond interactions; green lines indicate π–π interactions.

The latter results suggest that the extracted derived compounds, particularly gyrophoric acid and stictic acid, possess the structural requirements to interact effectively with both cholinesterases, potentially explaining the pharmacological profile of the total extracts. Figure 9 presents a two-dimensional representation of the principal interactions of the two top-performing derivatives, gyrophoric acid and stictic acid, at the catalytic sites of the enzymes where they exhibited their most favorable binding energies, namely acetylcholinesterase and butyrylcholinesterase, respectively.

The inhibitory potential of these compounds complements the biological benefits that have been previously reported. Gyrophoric acid is a tripdepside-type secondary metabolite with potent cytotoxic activity in cancer cell lines via the oxidative stress and apoptosis pathway [41], a photoprotective effect through the regulation of gene expression related to collagen protein [42], along with other biological activities such as antidiabetic, antihypertensive, antimicrobial, antiproliferative, and larvicidal effects [43]. For its part, stictic acid, an aromatic depsidone-type compound, possesses antimicrobial and cytotoxic activity [19] and, notably, antioxidant activity through increased cell viability and reduced production of reactive oxygen species [44,45]. It is important to note that although the extract’s inhibitory effect on acetylcholinesterase and butyrylcholinesterase was moderate, the identification of high-affinity constituents such as gyrophoric and stictic acids through molecular docking analysis suggests that the neuroprotective potential of the two lichen species lies in specific metabolites that should be isolated and purified in future studies.

### 2.5. Study Limitations

This study is the first report on the phytochemical composition and biological potential of the lichen species *P. candelaria* and *P. antarctica*; however, some limitations should be noted: first, the identification of the compounds present in the hydroalcoholic extracts is considered provisional, as it was based on high-resolution mass spectrometry and fragmentation patterns; therefore, in future studies, specific compounds will be isolated and their structures confirmed using nuclear magnetic resonance, and reference standards will also be used. Second, although molecular docking analyses provided a rational basis for understanding enzymatic activity, these in silico models do not account for processes such as bioavailability, blood–brain barrier permeability, or the metabolic transformation of the compounds; therefore, in future studies, it is important to use cytotoxicity assays and in vivo models to elucidate the safety and therapeutic efficacy of Antarctic lichen extracts. Third, the collected samples are limited to a specific geographic area; therefore, potential metabolic variability due to factors such as species distribution was not evaluated.

## 3. Materials and Methods

### 3.1. Lichen Material

The samples used in this study were collected in the South Shetland Islands archipelago, in Maritime Antarctica. A total of 50 g of *Polycauliona candelaria* were collected on Ardley Island in February 2024 (S 62°12′35.6″; W 58°55′58.4″), while 100 g of *Placopsis antarctica* were collected on Livingston Island in February 2025 (S 62°38.4401′ W 60°21.5868′); both collections were conducted during the Antarctic summer. Subsequently, both specimens were stored at the Natural Products Laboratory of the Institute of Pharmacy, Universidad Austral de Chile (Valdivia, Chile), under identification codes 602025 for *P. candelaria* and 612025 for *P. antarctica*.

### 3.2. Preparation of Hydroalcoholic Extract

Using 10 g of *P. candelaria* and *P. antarctica*, a hydroalcoholic extraction was performed using a methanol-water mixture (50:50 *v*/*v*), assisted by continuous sonication (80 kHz) for 30 min. The extract was then separated from the solid material by vacuum filtration. The filtrate was concentrated using a rotary evaporator at 40 °C under pressures ranging from 100–200 mbar. Subsequently, the samples were frozen at −80 °C and lyophilized to obtain dry extracts, which were then stored under dark, low-temperature conditions.

### 3.3. LC Parameters and MS Parameters

The metabolic profile characterization of the extracts was carried using an ultra-high-performance liquid chromatography system coupled to a quadrupole time-of-flight mass spectrometer with electrospray ionization (UHPLC-ESI-QToF-MS), specifically a Bruker Compact QToF instrument. For the analysis, 1.1 mg of *P. candelaria* and 1.3 mg of *P. antarctica* were weighed and suspended in 200 µL of 80% methanol by vortex mixing followed by ultrasound-assisted extraction for 10 min; after centrifugation at 12,000 *g* for 10 min, the supernatant was analyzed using a Kinetex C18 column (2.1 mm × 100 mm, 1.7 µm). Separation was performed at a flow rate of 0.4 mL/min and a column temperature of 40 °C, while samples were maintained at 8 °C in the autosampler; the mobile phase consisted of water with 0.1% formic acid as solvent A and 90% acetonitrile with 0.1% formic acid as solvent B, following a linear gradient starting at 12% B and reaching 99% B at 11 min, which was held for 3 additional minutes before re-equilibration. Detection was carried out using an ESI source in negative ion mode, with a capillary voltage of 4000 V, nebulizer pressure of 2.5 bar, and dry gas flow of 8.0 L/min at 230 °C, covering a scan range from 50 to 1500 *m*/*z*; fragmentation was obtained by Auto MS/MS with a collision energy of 7.0 eV and a stepping range from 100% to 250%, ensuring mass accuracy through the injection of sodium formate as an internal calibrant at the beginning of each run. Data processing and compound identification were performed using Metaboscape 4.0 software, applying annotation quality (AQ) criteria that considered a mass deviation of <20 ppm, optimal isotopic pattern matching (mSigma), and a similarity greater than 85% with MS/MS spectra from the RIKEN library. When no database matches were obtained, molecular formulas were determined using the SmartFormula algorithm.

### 3.4. Total Phenolic Content

The total phenolic content (TPC) was determined using the Folin–Ciocalteu colorimetric method [46]. A calibration curve was prepared using gallic acid as a standard by preparing a 1000 µg/mL stock solution and performing serial dilutions in ethanol to obtain concentrations of 25, 50, 75, 100, and 125 µg/mL. The extracts of *P. candelaria* and *P. antarctica* were prepared at a concentration of 1000 µg/mL in ethanol. The assay was carried out in a transparent 96-well microplate in triplicate, adding 10 µL of sample, 150 µL of deionized water, 12.5 µL of 10% Folin–Ciocalteu reagent, and 37.5 µL of 20% sodium carbonate (Na_2_CO_3_). The mixture was incubated for 30 min at room temperature in the dark, and the absorbance was subsequently measured at 765 nm using a microplate reader. The analysis was performed using the following curve equation and coefficient of determination: y = 0.002x + 0.0189 and R^2^ = 0.9915.

### 3.5. Ferric-Reducing Antioxidant Power (FRAP) Assay

The antioxidant capacity by the FRAP (Ferric Reducing Antioxidant Power) assay was based on the reduction in the ferric complex of 2,4,6-tris(2-pyridyl)-s-triazine (Fe^3+^-TPTZ) to its ferrous form (Fe^2+^-TPTZ) [47]. For this purpose, a FRAP working solution was prepared consisting of 12 mM acetate buffer (pH 3.6), 10 µM TPTZ in ethanol, and 20 mM ferric chloride (FeCl_3_·6H_2_O). For sample quantification, a Trolox calibration curve was constructed from a 1000 µg/mL stock solution, from which concentrations of 800, 600, 400, 200, 120, 60, and 40 µg/mL in ethanol were prepared. The extracts of *P. candelaria* and *P. antarctica* were prepared at a concentration of 1000 µg/mL in ethanol. The assay was performed in a 96-well microplate in triplicate, adding 10 µL of standard or sample, followed by 290 µL of the FRAP working solution. The mixture was incubated for 30 min at room temperature in the dark, and the absorbance was measured at 593 nm using a microplate reader. The analysis was performed using the following curve equation and coefficient of determination: y = 0.0001x + 0.0508 and R^2^ = 0.9918.

### 3.6. Oxygen Radical Absorbance Capacity (ORAC) Assay

The peroxyl radical scavenging activity was evaluated using the ORAC (Oxygen Radical Absorbance Capacity) assay [48]. For this purpose, 75 mM phosphate-buffered saline (PBS, pH 7.4), fluorescein at 108 nM, and 2,2′-azobis(2-methylpropionamidine) dihydrochloride (AAPH) at 18 mM were used, with all solutions protected from light during the procedure. The extracts of *P. candelaria* and *P. antarctica* were prepared at an initial concentration of 1000 µg/mL in PBS, from which dilutions of 500, 50, and 5 µg/mL were obtained. The assay was performed in a black 96-well microplate in triplicate, adding 45 µL of each sample dilution followed by 175 µL of fluorescein to all wells. The microplate was incubated for 30 min at 37 °C in the dark, after which 50 µL of AAPH was added, and the kinetic reading was immediately started for 1 h and 30 min, recording fluorescence every 2 min at an excitation wavelength of 480 nm and emission at 520 nm using a microplate reader. The analysis was performed using the following curve equation and coefficient of determination: y = 0.8701x + 6.4887 and R^2^ = 0.9862.

### 3.7. DPPH Radical Scavenging Assay

The free radical scavenging activity was evaluated using the assay based on the reduction in the 2,2-diphenyl-1-picrylhydrazyl radical (DPPH•) [49]. For this purpose, a DPPH• working solution at 156 µM was prepared in an ethanol–water mixture (50:50 *v*/*v*) and kept protected from light throughout the procedure. The extracts of *P. candelaria* and *P. antarctica* were prepared at an initial concentration of 1000 µg/mL in the same solvent system, from which serial dilutions were made to obtain concentrations of 1000, 500, 250, 125, 62.5, 31.25, 15.6, and 7.8 µg/mL. The assay was performed in a 96-well microplate in triplicate, adding 50 µL of each sample dilution followed by 150 µL of the DPPH• solution to all wells. The microplate was incubated for 30 min in the dark at room temperature, and the absorbance was finally measured at 517 nm using a microplate reader. The analysis was performed using the following curve equations and coefficients of determination: y = 0.0333x + 13.13 (R^2^ = 0.9586) for *P. candelaria*, and y = 0.0752x + 25.136 (R^2^ = 0.9542) for *P. antarctica*.

### 3.8. ABTS Radical Scavenging Assay

The antioxidant activity was evaluated using the ABTS•^+^ radical reduction assay [50]. For this purpose, an ABTS•^+^ working solution was prepared in an aqueous medium, consisting of 7 mM ABTS and 3.6 mM potassium persulfate (K_2_S_2_O_8_), which was kept in the dark for 24 h and subsequently diluted to a concentration of 169 µM in deionized water. The extracts of *P. candelaria* and *P. antarctica* were prepared at an initial concentration of 1000 µg/mL, from which serial dilutions were performed to obtain concentrations of 1000, 500, 250, 125, 62.5, 31.25, 15.6, and 7.8 µg/mL. The assay was carried out in a 96-well microplate in triplicate, adding 50 µL of each sample dilution followed by 150 µL of the ABTS•^+^ working solution to all wells. The microplate was incubated for 30 min in the dark at room temperature, and the absorbance was finally measured at 732 nm using a microplate reader. The analysis was performed using the following regression equation and coefficient of determination for *P. candelaria* and *P. antarctica*, respectively: y = 0.1111x + 18.065, with R^2^ = 0.9907; and y = 0.6194x + 10.775, with R^2^ = 0.9709.

### 3.9. Cholinesterase Inhibition

Acetylcholinesterase (AChE) inhibition was evaluated [51] using a 50 mM Tris-HCl buffer (pH 8), 5,5′-Dithiobis(2-nitrobenzoic acid) (DTNB) at 3 mM, and acetylcholine at 5 mM as substrate. The AChE enzyme was prepared at a working concentration of 0.28 U/mL in buffer. The extracts of *P. candelaria* and *P. antarctica* were prepared at an initial concentration of 4000 µg/mL in buffer, from which serial dilutions were made to obtain concentrations of 2000, 1500, 250, 125, 62.5, and 31.25 µg/mL. The assay was performed in a 96-well microplate in triplicate, adding 25 µL of sample, 125 µL of DTNB, and 25 µL of enzyme. The microplate was incubated for 15 min at 37 °C, after which 25 µL of the acetylcholine substrate was added. Subsequently, it was incubated for an additional 10 min at 37 °C, and the absorbance was recorded in kinetic mode at 412 nm using a microplate reader. The analysis was performed using the following curve equations and coefficients of determination: y = 0.0759x + 14.2 (R^2^ = 0.9552) for *P. candelaria*, and y = 0.0462x + 19.766 (R^2^ = 0.9698) for *P. antarctica*.

Additionally, the inhibition of butyrylcholinesterase (BuChE) [51] was evaluated using a 50 mM Tris-HCl buffer at pH 8, 3 mM DTNB reagent, and 5 mM butyrylcholine as substrate. The BuChE enzyme was prepared at a working concentration of 0.28 U/mL in buffer. Extracts of *P. candelaria* and *P. antarctica* were prepared at an initial concentration of 4000 µg/mL in buffer, from which serial dilutions were made to obtain concentrations of 2000, 1000, 500, 250, 125, 62.5, and 31.25 µg/mL. The assay was carried out in a 96-well microplate in triplicate by adding 25 µL of sample, 125 µL of DTNB, and 25 µL of enzyme. The microplate was incubated for 15 min at 37 °C, after which 25 µL of the butyrylcholine substrate was added. Subsequently, it was incubated for an additional 10 min at 37 °C, and the absorbance was measured at 412 nm in kinetic mode using a microplate reader. The analysis was performed using the following curve equations and coefficients of determination for *P. candelaria* and *P. antarctica*, respectively: y = 0.0333x + 13.13 (R^2^ = 0.9530) and y = 0.0398x + 16.346 (R^2^ = 0.9625).

### 3.10. Docking Simulations

Docking simulations were performed for gluconic acid, orsellinic acid, stictic acid, and gyrophoric acid from the *P. antarctica* extract. For the *P. candelaria* extract, emodin and linoleic acid were analyzed. All compounds are shown in Figure 6. Energetic minimization of each molecule were carried out using the LigPrep tool in Maestro Schrodinger suite v.11.8 (Schrödinger, LLC, New York, NY, USA) [52]. The crystallographic structure of *Torpedo californica* acetylcholinesterase (*Tc*AChE; PDB ID:1DX6 [53]) and human butyrylcholinesterase (*h*BuChE; PDBID:4BDS [54]) were obtained from the RCSB Protein Data Bank [55]. Enzyme optimization was carried out using Maestro’s Protein Preparation Wizard. All water molecules located in the catalytic pocket and throughout the enzyme were deleted prior to further processing. Protonation states of acidic and basic residues and polar hydrogen atoms were determined at physiological pH (7.4). The enclosing box size was set to a cube with sides of 26 Å length and the OPLS3e force field was used to minimize protein energy. The grid centroids were assigned to the putative catalytic sites of the enzymes, based on the positions of the catalytic residues and the bound galantamine or tacrine in the crystal enzyme structures of *Tc*AChE and *h*BuChE, respectively. The Glide Induced Fit Docking protocol has been used for the final couplings [56]. All derivatives were scored using the Glide XP (extra-precision) scoring function (Schrödinger, LLC) [57] and filtered based on docking scores and RMSD values (cutoff < 1.0 Å) to identify favorable ligand–enzyme interactions, binding modes, and contact clashes. The scoring value of galantamine within acetylcholinesterase was selected based on comparison with the inhibitor observed in the 1DX6 crystal structure. In contrast, for butyrylcholinesterase, the scoring value of galantamine was determined based on its favorable ligand–enzyme interactions. Resulting docking complexes were visually inspected using Visual Molecular Dynamics (VMD) and PyMOL v. 3.1.8 [58].

### 3.11. Statistical Analysis

Extracts of each lichen were prepared from a homogeneous mixture of several subsamples collected at different locations within the same area. For the in vitro assays of TPC, FRAP, ORAC, DPPH, ABTS, AChE, and BuChE, each measurement was performed in three independent experiments, with samples analyzed in triplicate. The results were presented as the mean ± standard deviation (SD) using Microsoft Excel 2019 (Microsoft Office, Microsoft Corporation, Redmond, WA, USA). To compare the means, a one-way analysis of variance (ANOVA) was performed, followed by Tukey’s test with a significance level of *p* < 0.05, using GraphPad Prism 8 (GraphPad Software Corporation, La Jolla, CA, USA).

## 4. Conclusions

This study enabled the characterization of the phytochemical profiles of hydroalcoholic extracts from the Antarctic lichen species *Polycauliona candelaria* and *Placopsis antarctica*, as well as the evaluation of their antioxidant and enzyme-inhibitory activities. UHPLC-ESI-QToF-MS analysis revealed the presence of various compounds in both species, including, in particular, fatty acids, phenolic derivatives, depsides, depsidones, dibenzofurans, and anthraquinones, which further underscore the chemical richness of lichens from polar regions. On the other hand, in vitro assays of antioxidant activity and cholinesterase inhibition showed variable and moderately significant potential for both species. Complementarily, in silico analyses indicated that the compounds known as gyrophoric acid, stictic acid, and emodin possess the highest affinity for the catalytic sites of the enzymes acetylcholinesterase and butyrylcholinesterase.

These findings enhance our understanding of the potential of bioactive lichen compounds in studying their therapeutic effects on reducing oxidative stress and cholinergic deficit in models of neurodegenerative diseases. However, this study is preliminary in nature, and future research should continue with the isolation and purification of compounds, as well as expand preclinical in vitro and in vivo studies to elucidate cytotoxicity, dose–response effects, and mechanisms of action, thereby providing evidence to support their safety and efficacy for future clinical trials.

Furthermore, it is important to note that this study represents the first report on the chemical composition and biological activities of the species *P. candelaria* and *P. antarctica*, and is part of an ongoing multidisciplinary effort to understand and assess Antarctic lichen communities. Unlike other studies on Antarctic lichens, this study is notable for its focus on characterizing their hydroalcoholic extracts; furthermore, in the study of molecular docking with cholinesterase enzymes, it examined other compounds that had not been previously evaluated.

## Figures and Tables

**Table 1:** Identification by UHPLC/ESI/QToF/MS of the compounds present in the hydroalcoholics extract of *P. candelaria*.

Peak	Retention Time (min)	Tentative Identification	Molecular Formula	Theoretical Mass ([M − H]^−^ *m*/*z*)	Measured Mass ([M − H]^−^ *m*/*z*)	Accuracy (ppm)	Metabolite Type	MS/MS Ions
1	0.6	2-Isopropylmalic acid	C_7_H_11_O_5_	181.0718	181.0686	17.6	Organic acid	163.0039
2	4.0	Mannitol	C_6_H_13_O_6_	191.05501	191.05592	3.77	Sugar alcohol	127.03968
3	4.7	Nitrogenous derivative	C_13_H_9_N_2_O_4_	257.0568	257.0598	11.6	-	197.0724
4	4.9	Nitrogenous derivative	C_14_HN_2_O_8_	325.1663	324.9636	11	-	112.9842, 321.4077
5	5.0	3-Acetyl benzoic acid	C_9_H_7_O_3_	163.0401	163.0375	15.7	Phenolic derivative	112.9839
6	5.3	Nitrogenous derivative	C_14_HN_2_O_8_	325.1663	324.9627	11	-	112.9842, 321.4077
7	5.7	Nitrogenous derivative	C_14_HN_2_O_8_	324.9737	324.9645	−2.4	-	260.9927, 747.3746
8	6.5	Emodin	C_15_H_9_O_5_	269.0407	269.0409	0.7	Anthraquinone	112.9841, 225.05614
9	7.1	13-Hydroxy-8′-apo-beta-caroten-8′-al	C_30_H_39_O_2_	431.3028	431.3103	−17.5	Carotenoid	112.9839
10	7.6	Hexadecatetraenoic acid	C_16_H_23_O_2_	247.1704	247.1664	17.3	Fatty acid	175.1480
11	8.1	Usnic acid	C_18_H_15_O_7_	343.08123	343.0779	1.16	Dibenzofuran	-
12	8.8	Usnic acid isomer	C_18_H_15_O_7_	343.08123	343.0781	1.16	Dibenzofuran	-
13	9.6	Alpha linoleic acid	C_18_H_29_O_2_	277.2173	277.2131	14.6	Fatty acid	259.0572
14	10.5	Linoleic acid	C_18_H_31_O_2_	279.2330	279.2284	16.3	Fatty acid	112.9837
15	11.3	Palmitic acid	C_16_H_31_O_2_	255.2329	255.2289	16.3	Fatty acid	116.9267
16	11.6	Oleic acid	C_18_H_33_O_2_	281.2486	281.2442	15.3	Fatty acid	116.9264

**Table 3:** Total phenolic content (TPC) and antioxidant activity of the hydroalcoholic extracts of lichens *P. candelaria* and *P. antarctica*.

Assay	TPC mg GAE/g	FRAP µmol Trolox/g	ORAC µmol Trolox/g	DPPH IC_50_-µg/mL	ABTS IC_50_-µg/mL
*P. candelaria*	0.56 ± 0.02 *	46.60 ± 1.20 *	409.60 ± 2.40 *	2344.13 ± 0.02 *	287.44 ± 0.02 *
*P. antarctica*	2.69 ± 0.15 *	24.60 ± 3.60 *	513.70 ± 8.80 *	330.64 ± 0.02 *	63.33 ± 0.02 *
Gallic acid ^#^	-	-	-	2.24 ± 0.04 *	16.50 ± 0.04 *
Quercetin ^#^	-	-	-	12.25 ± 0.60 *	15.65 ± 0.05 *

Values marked with * are statistically different (*p* < 0.05). ^#^ Positives controls.

**Table 4:** Enzyme inhibition activity of the hydroalcoholic extracts of lichens *P. candelaria* and *P. antarctica*.

Assay	AChE (IC_50_-µg/mL)	BuChE (IC_50_-µg/mL)
*P. candelaria*	471.67 ± 0.04 *	1107.28 ± 0.01 *
*P. antarctica*	654.42 ± 0.03 *	845.58 ± 0.01 *
Galantamine ^#^	0.27 ± 0.03 *	3.82 ± 0.03 *

Values marked with * are statistically different (*p* < 0.05). ^#^ Positives controls.

**Table 5:** Binding energies obtained from docking experiments of selected compounds from the *P. antarctica* and *P. candelaria* extracts, as well as the known inhibitor galantamine over acetylcholinesterase (*Tc*AChE), and butyrylcholinesterase (*h*BuChE).

Compound	Binding Energy (kcal/mol) Acetylcholinesterase	Binding Energy (kcal/mol) Butyrylcholinesterase
Gluconic acid	−8.833	−7.829
Orsellinic acid	−7.705	−5.918
Stictic acid	−11.105	−10.529
Gyrophoric acid	−11.734	−8.777
Emodin	−11.521	−9.470
Linoleic acid	−7.348	−4.527
Galantamine	−11.749	−7.458

## Data Availability

The datasets presented in this study can be consulted with the authors by correspondence.

## Supplementary Materials

The following supporting information can be downloaded at: https://www.mdpi.com/article/10.3390/molecules31132242/s1, Figure S1: Mass spectrum of the compound emodin found in *P. candelaria*; Figure S2: Mass spectrum of the compound linoleic acid found in *P. candelaria*; Figure S3: Mass spectrum of the compound gluconic acid found in *P. antarctica*; Figure S4: Mass spectrum of the compound orsellinic acid found in *P. antarctica*; Figure S5: Mass spectrum of the compound stictic acid found in *P. antarctica*; Figure S6: Mass spectrum of the compound gyrophoric acid found in *P. antarctica*.
