# Supplementary material for: Phytochemical Profiling and Antioxidant and Enzymatic Evaluation of Extracts from the Antarctic Lichens Polycauliona candelaria and Placopsis antarctica
Source: Molecules. 2026 Jun 25;31(13):2242. doi: 10.3390/molecules31132242 (PMC13362635; doi:10.3390/molecules31132242)
Supplement: Supplementary file 1 [file molecules-31-02242-s001.zip › molecules-4327050-supplementary.pdf]

Supplementary Information

Phytochemical Profiling and Antioxidant and Enzymatic Evaluation of Extracts from the Antarctic Lichens *Polycauliona candelaria* and *Placopsis antarctica*

Alfredo Torres-Benítez [1,*], Nicolás Pizarro-Piña [2], Javier Romero-Parra [3], Gabriel Vargas-Arana [4], Marta Sánchez [5], María Pilar Gómez-Serranillos [5] and Mario J. Simirgiotis [2,*]

[1] Carrera de Química y Farmacia, Facultad de Ciencias, Universidad San Sebastián, General Lagos 1163, Valdivia 5090000, Chile
[2] Instituto de Farmacia, Facultad de Ciencias, Universidad Austral de Chile, Campus Isla Teja, Valdivia 5090000, Chile; nicolas.pizarro01@alumnos.uach.cl
[3] Departamento de Química Orgánica y Fisicoquímicas, Facultad de Ciencias Químicas y Farmacéuticas, Universidad de Chile, Olivos 1007, Santiago 8380544, Chile; javier.romero@ciq.uchile.cl
[4] Facultad de Industrias Alimentarias, Universidad Nacional de la Amazonía Peruana, Iquitos 16001, Peru; gabriel.vargas@unapiquitos.edu.pe
[5] Departamento de Farmacología, Farmacognosia y Botánica, Facultad de Farmacia, Universidad Complutense de Madrid, Plaza Ramón y Cajal s/n, Ciudad Universitaria, 28040 Madrid, Spain; martas15@ucm.es (M.S.); pserra@ucm.es (M.P.G.-S.)
* Correspondence: alfredo.torres@uss.cl (A.T.-B.); mario.simirgiotis@uach.cl (M.J.S.)

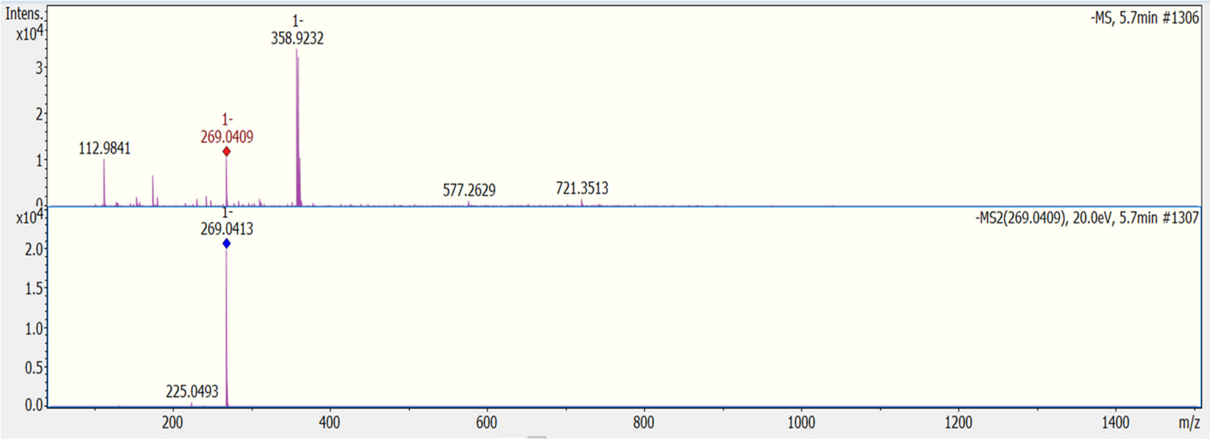

Figure S1. Mass spectrum of the compound emodin found in *P. candelaria.*

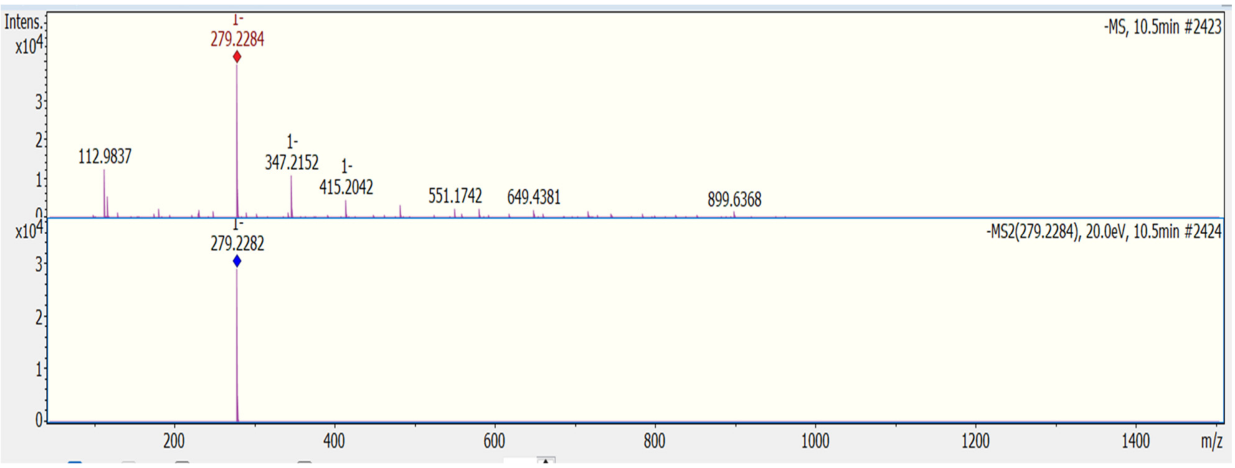

Figure S2. Mass spectrum of the compound linoleic acid found in *P. candelaria.*

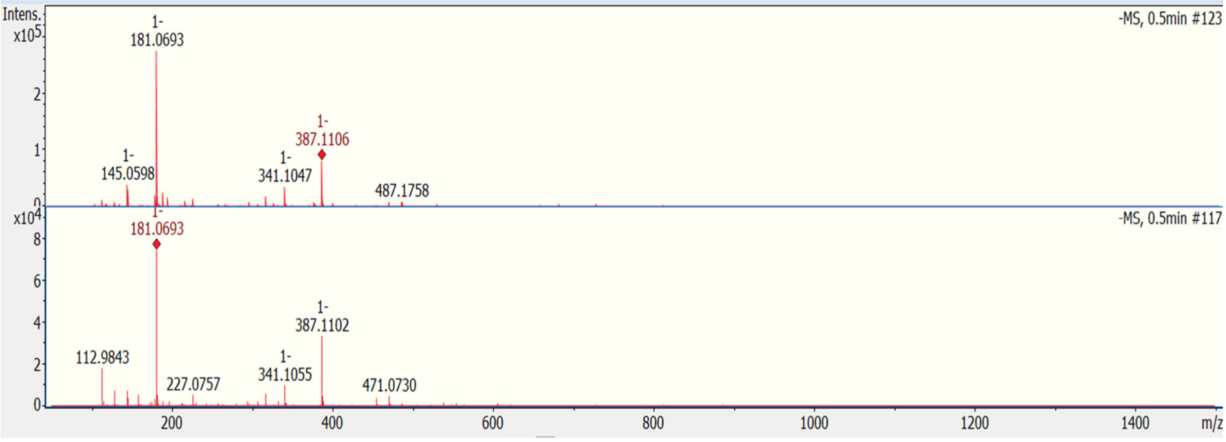

Figure S3. Mass spectrum of the compound gluconic acid found in *P. antarctica.*

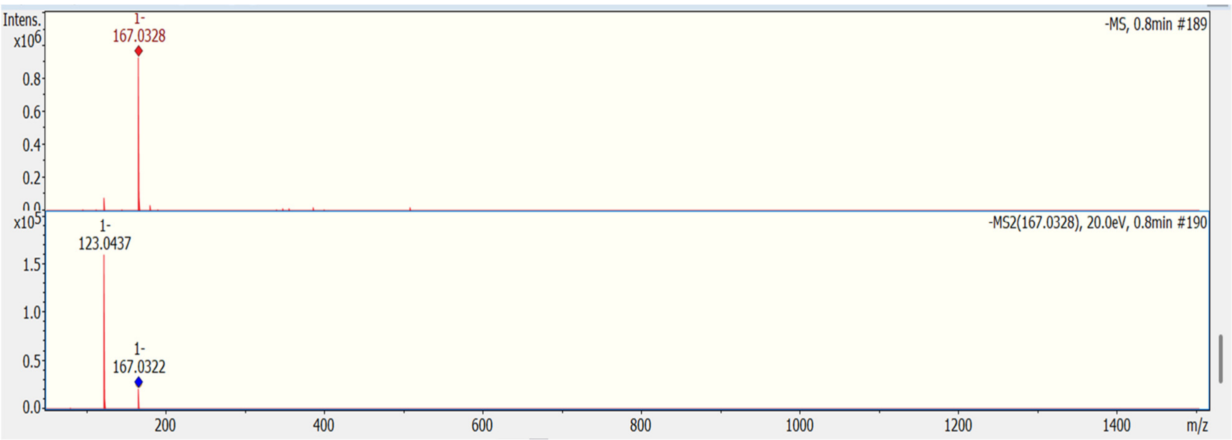

Figure S4. Mass spectrum of the compound orsellinic acid found in *P. antarctica.*

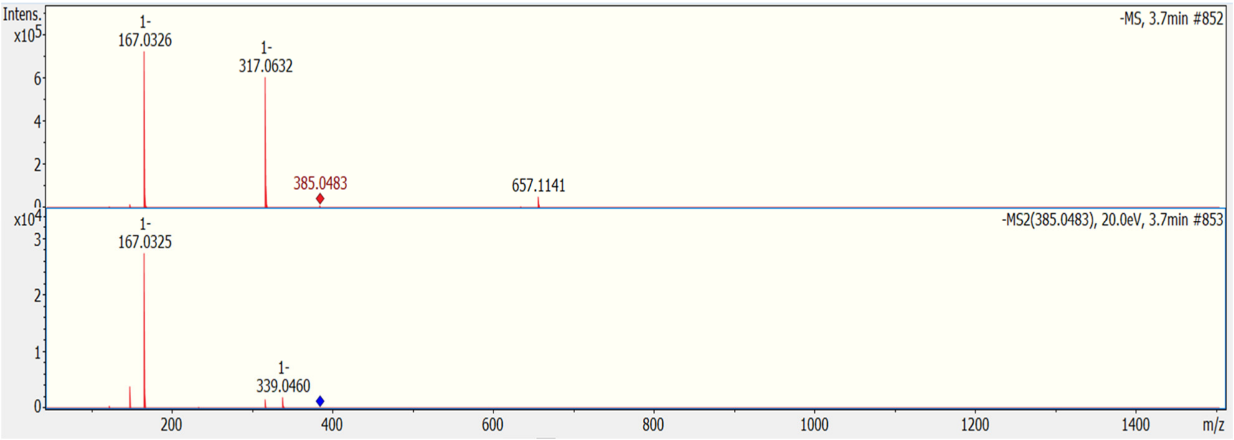

Figure S5. Mass spectrum of the compound stictic acid found in *P. antarctica.*

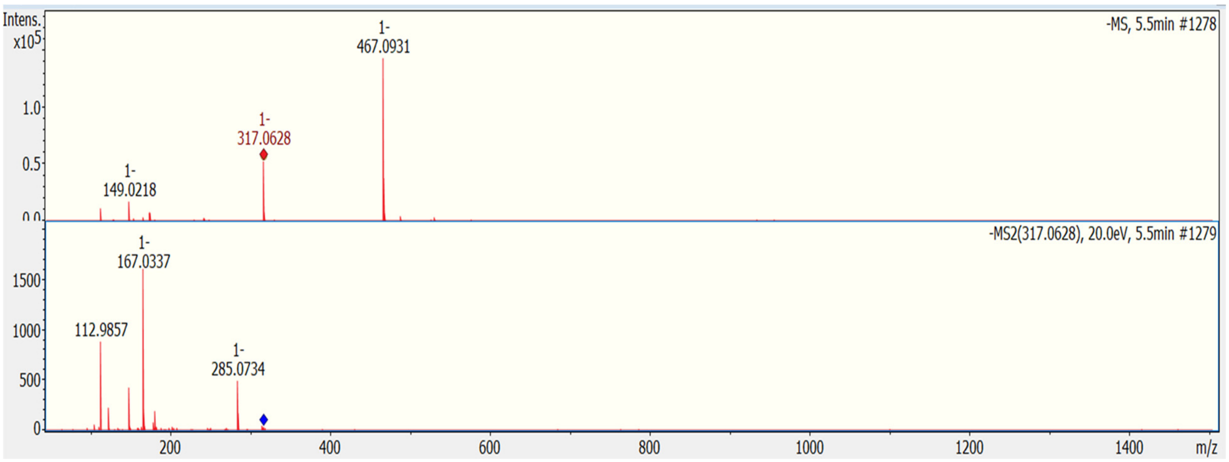

Figure S6. Mass spectrum of the compound gyrophoric acid found in *P. antarctica.*
